# Fish Intake May Affect Brain Structure and Improve Cognitive Ability in Healthy People

**DOI:** 10.3389/fnagi.2020.00076

**Published:** 2020-03-20

**Authors:** Keisuke Kokubun, Kiyotaka Nemoto, Yoshinori Yamakawa

**Affiliations:** ^1^Open Innovation Institute, Kyoto University, Kyoto, Japan; ^2^Division of Clinical Medicine, Department of Neuropsychiatry, Faculty of Medicine, University of Tsukuba, Tsukuba, Japan; ^3^ImPACT Program of Council for Science, Technology and Innovation (Cabinet Office, Government of Japan), Chiyoda, Tokyo, Japan; ^4^Institute of Innovative Research, Tokyo Institute of Technology, Meguro, Tokyo, Japan; ^5^Office for Academic and Industrial Innovation, Kobe University, Kobe, Japan; ^6^NTT Data Institute of Management Consulting, Inc., Chiyoda, Tokyo, Japan

**Keywords:** fractional-anisotropy brain healthcare quotient, gray-matter brain healthcare quotient, magnetic resonance imaging data, MCI Screen, brief self-administered diet history questionnaire, fish intake

## Abstract

As the population ages worldwide, the prevalence of cognitive disorders including mild cognitive impairment (MCI) is increasing. MCI appears in 10–20% of adults aged 65 years and older and is generally referred to as an intermediate stage between normal cognitive aging and dementia. To develop timely prevention and early treatment strategies by identifying biological factors, we investigated the relationship between dietary consumption of fish, brain structure, and MCI in cognitively normal subjects. The brain structure was assessed using neuroimaging-derived measures including the “gray-matter brain healthcare quotient (GM-BHQ)” and “fractional-anisotropy brain healthcare quotient (FA-BHQ),” which are approved as the international standard (H.861.1) by the International Telecommunication Union Telecommunication Standardization Sector. Dietary consumption of fish was calculated using the brief self-administered diet history questionnaire (BDHQ), and MCI was assessed using the Memory Performance Index (MPI) of MCI screening method (MCI Screen). This study showed that fish intake was positively associated with both FA-BHQ and MPI, and FA-BHQ was more strongly associated with MPI than fish intake. Our findings are in line with those in previous studies, but our study further indicates that the condition of the whole brain integrity measured by the FA-BHQ may mediate the relationship between fish intake and MCI prevention in healthy people. In other words, FA-BHQ may be used to identify people at high risk of MCI to provide the appropriate intervention.

## Introduction

As the population ages worldwide, the prevalence of cognitive disorders, including mild cognitive impairment (MCI), is growing (Alzheimer’s Disease International, 2015). MCI appears in 10–20% of adults aged 65 years and older (Langa and Levine, 2014) and is generally referred to as an intermediate stage between normal cognitive aging and dementia (Vega and Newhouse, 2014). Individuals with MCI are 6.7 times more likely to have Alzheimer’s disease (AD) than cognitively normal individuals (Boyle et al., 2006). However, no medications have proven effective for MCI currently (Langa and Levine, 2014). Therefore, identification of biological factors that may be implicated in MCI is essential for timely prevention and early treatment.

Fish is an important source of omega-3 fatty acids (n-3 FAs) that are present in the membranes of the brain tissue (Weiser et al., 2016); therefore, a fish-oriented dietary intake has been increasingly focused on to investigate the role of nutrition in the prevention of cognitive disorders. Indeed, it was found that a higher intake of n-3 FAs or total polyunsaturated fatty acids was associated with a lower risk of MCI (Devore et al., 2009; Zhang et al., 2015), probably because eicosapentaenoic acid and docosahexaenoic acid (DHA) are protective factors in the nervous system in humans (Calon and Cole, 2007; Kou et al., 2008). In addition, fishery products are recommended as dietary sources because fish consumption has been found to be associated with a reduced risk of dementia (Huang et al., 2005) or AD (Zhang et al., 2015).

However, some studies have shown that fish consumption does not have a protective effect on MCI. A meta-analysis by Zeng et al. (2017) found no statistically significant association between fish intake and the risk of MCI. The inconsistency in the association between fish intake and MCI may be due to indirectness. In other words, the brain, the mediator between fish consumption and cognitive ability, could be influenced by many unpredictable biological and sociological factors other than fish intake; therefore, a direct and significant relationship between fish intake and MCI might be difficult to be observed. Therefore, understanding the effect of fish consumption on brain structure, rather than on MCI, is critical for the determination of modifiable factors that can decrease the risk of cognitive deficits and dementia by intervention earlier in life (Lopez et al., 2013). Notably, a previous study found that daily fish oil supplementation increased the gray matter (GM) volume and decreased the white matter lesions (WMLs) in cognitively normal people (Witte et al., 2013); another study demonstrated that lower DHA levels in red blood cells were associated with lower total brain volume and higher WML volumes (Bowman et al., 2012; Tan et al., 2012). It has been demonstrated that a member of the major facilitator superfamily transporters, i.e., major Facilitator Superfamily Domain Containing 2A (Mfsd2a), previously an orphan transporter, is involved in the DHA uptake into the brain (Nguyen et al., 2014). In addition, other previous studies also indicate the link between brain structure and MCI. For instance, one previous study found significant differences in the white matter integrity between MCI patients with and without cerebral amyloidopathy, indicating that alteration in white matter integrity can serve as a potential biomarker of MCI (Lee et al., 2017). In the same vein, several studies indicate that WML is associated with cognitive decline and incident dementia (Bokura et al., 2006; Buyck et al., 2009; Debette et al., 2010; Debette and Markus, 2010). Moreover, WML and cognitive deficits have been reported in various animal models contributing to the mechanism clarification (Hainsworth et al., 2012; Prins and Scheltens, 2015). For instance, in a mouse model of vascular dementia, astroglial nuclear factor-kB contributed to white matter damage and cognitive impairment (Saggu et al., 2016).

Therefore, this study aimed to investigate the relationships between dietary consumption of fish and brain structure as well as between brain structure and MCI in cognitively normal subjects. We examined the brain structure using the neuroimaging-derived measures, including the gray-matter brain healthcare quotient (GM-BHQ) and the fractional-anisotropy brain healthcare quotient (FA-BHQ), which are approved as the international standard (H.861.1) by International Telecommunication Union Telecommunication Standardization Sector (ITU-T). The fish intake volume was calculated using the brief self-administered diet history questionnaire (BDHQ; Sasaki et al., 2000), and MCI was assessed using the Memory Performance Index (MPI) of MCI screening method (MCI Screen; the Medical Care Corporation). The tested hypotheses are presented as follows: (1) the frequency of fish consumption correlates with higher FA-BHQ (and/or GM-BHQ) scores; (2) The fish consumption frequency and the FA-BHQ (and/or GM-BHQ) scores independently correlate with MPI, and the FA-BHQ (and/or GM-BHQ) scores have a stronger association with MPI than the fish consumption frequency.

## Materials and Methods

### Subjects

Eighty-four healthy participants (47 females and 37 males) were recruited in Kyoto, Japan, in May to June 2017, with support of a personnel service company. Potential participants who had a history of neurological, psychiatric, or medical conditions that could affect the central nervous system were excluded from the study. We administered the three items of “Depression,” a subscale of the NEO-Five Factor Inventory (Costa and McCrae, 1992), to screen for depression. Eight participants whose Depression scores were higher than 9 points were excluded from this study because they might be suffering from depression. Finally, the study included 76 participants (45 females and 31 males), aged 31–59 years [mean (M) ± standard deviation (SD): 47.0 ± 7.1 years]. The average age of participants was lower than other prior experiments regarding MCI (c.f., Langa and Levine, 2014) because the purpose of the current study is to clarify the association between brain structure and “cognitive ability in healthy people.” This study was approved by the Ethics Committees of Kyoto University (approval number 27-P-13) and was performed in accordance with the guidelines and regulations of the institute. All participants provided written informed consent before participation, and participant anonymity has been preserved.

### Fish Intake Scale

We employed the following two of the four fish dietary habit questions, which are included in the validated BDHQ (Sasaki et al., 2000), to assess the frequency of fish intake: “how often have you eaten grilled fish during the preceding month?” and “how often have you eaten tempura or fried fish during the preceding month?” Participants were requested to respond to the following items on a 7-point scale: (1) 2 times and more daily, (2) 1 time daily, (3) 4–6 times weakly, (4) 2–3 times weakly, (5) 1 time weakly, (6) less than 1 time weakly, and (7) did not eat. The amount of fish consumed (gram) was calculated from the combined figure of these items by a computer algorithm using the Standard Tables of Food Composition in Japan (Science and Technology Agency, 2010). Hereafter, we call this figure “Fish Intake.” We did not include other two questions of the four fish dietary habit questions, which are related to “sashimi and sushi” and “boiled fish, stew, soup, and miso soup,” because we thought these two questions are ambiguous and might be differently perceived by different individuals.

### Assessment of MCI

MCI was assessed using the Japanese version of the MCI Screen. MCI Screen is a 10 min staff-administered test to assess memory, executive function, and language (Shankle et al., 2005); it was developed by the Medical Care Corporation (Irvine, CA, United States) based on the protocol of the Consortium to Establish a Registry for Alzheimer’s Disease 10-word recall test. The score of MPI is computed by the results of sequential tasks, including three immediate recall tasks, a triadic comparison task, a judgment task, a delayed free recall task, a cued-recall task, and a rehearsed recall task. MPI ranges from 0 to 100 and larger values indicate better performance. The score can be used to discriminate amnestic or mixed cognitive MCI from normal aging with a 97% accuracy rate (Shankle et al., 2005).

### MRI Data Acquisition

All magnetic resonance imaging (MRI) data were collected using a 3-T Siemens scanner (Verio, Siemens Medical Solutions, Erlangen, Germany or MAGNETOM Prisma, Siemens, Munich, Germany) with a 32-channel head array coil. A high-resolution structural image was acquired using a three-dimensional (3D) T1-weighted magnetization-prepared rapid-acquisition gradient echo (MP-RAGE) pulse sequence. The parameters were as follows: repetition time (TR), 1900 ms; echo time (TE), 2.52 ms; inversion time (TI), 900 ms; flip angle, 9°; matrix size, 256 × 256; field of view (FOV), 256 mm; and slice thickness, 1 mm. DTI data were collected with spin-echo echo-planar imaging (SE-EPI) with GRAPPA (generalized autocalibrating partially parallel acquisitions). The image slices were parallel to the orbitomeatal (OM) line. The parameters were as follows: TR, 14,100 ms; TE, 81 ms, flip angle, 90°; matrix size, 114 × 114; FOV, 224 mm; slice thickness, 2 mm. A baseline image (b = 0 s/mm^2^) and 30 different diffusion orientations were acquired with a b value of 1000 s/mm^2^.

### GM-BMQ and FA-BHQ

T1-weighted images were preprocessed and analyzed using Statistical Parametric Mapping 12 (SPM12; Wellcome Trust Centre for Neuroimaging, London, United Kingdom) running on MATLAB R2015b (Mathworks Inc., Sherborn, MA, United States). Each MPRAGE image was segmented into GM, white matter (WM), and cerebrospinal fluid (CSF) images. The segmented GM images were spatially normalized using the diffeomorphic anatomical registration through exponentiated lie algebra (DARTEL) algorithm (Ashburner, 2007). A modulation step was also incorporated into the preprocessing model to reflect regional volume and preserve the total GM volume from before the warp. As a final preprocessing step, all normalized, segmented, modulated images were smoothed with an 8 mm full width at half-maximum (FWHM) Gaussian kernel. Intracranial volume (ICV) was also calculated by summing the GM, WM, and CSF images for each subject. Proportional GM images were generated by dividing smoothed GM images by ICV to control for differences in whole-brain volume across participants. Using these proportional GM images, mean and standard deviation (SD) images were generated from all participants. Next, we calculated the GM brain healthcare quotient (BHQ), which is similar to the intelligence quotient (IQ). The mean value was defined as BHQ 100 and SD was defined as 15 BHQ points. By this definition, approximately 68% of the population is between BHQ 85 and BHQ 115, and 95% of the population is between BHQ 70 and BHQ 130. Individual GM quotient images were calculated using the following formula: 100 + 15 × (individual proportional GM–mean)/SD. Regional GM quotients were then extracted using an automated anatomical labeling (AAL) atlas (Tzourio-Mazoyer et al., 2002) and averaged across regions to produce participant-specific GM-BHQs.

DTI data were preprocessed using FMRIB Software Library (FSL) 5.0.11 (Jenkinson et al., 2012). First, all diffusion images were aligned with the initial b0 image, and motion correction and eddy current distortion correction was performed using eddy_correct. Following these corrections, FA images were calculated using dtifit. FA images were then spatially normalized into the standard Montreal Neurological Institute (MNI) space using FLIRT and FNIRT. After spatial normalization we smoothed the data with an 8-mm FWHM. Mean and SD images were generated from all the FA images, and individual FA quotient images were calculated using the following formula: 100 + 15 × (individual FA–mean)/SD. Regional FA quotients were extracted using Johns Hopkins University (JHU) DTI-based white-matter atlases (Mori et al., 2008) and averaged across regions to produce participant-specific FA-BHQs. For more details, please see Nemoto et al. (2017).

### Statistical Analysis

The hierarchical regression analysis was employed to assess the correlation between MPI, FA-BHQ, and Fish Intake. In the model with FA-BHQ scores as the dependent variables, we entered the control variables (including GM-BHQ scores) in Step 1 and Fish Intake in Step 2. In the model with MPI as the dependent variable, we entered the control variables in Step 1 and the main effects of FA-BHQ or Fish Intake in Step 2. In Step 3, we entered all the variables simultaneously. We added these variables to the models based on the following hypotheses: Fish Intake is closely related to FA-BHQ; FA-BHQ or Fish Intake is closely related to MPI; and FA-BHQ is more closely related to MPI than Fish Intake after adjusting for demographic information. Body mass index (BMI) was included in the model because obesity has been found to be associated with an approximately 50–70% increased risk of MCI (Wang et al., 2017). In addition, length of education was also included in the model because a 7-year period longitudinal study has revealed that the education level modulates the effects of WML on the risk of MCI (Mortamais et al., 2014), possibly because of stronger myelination and more richly connected fiber tracts in the white matter in highly educated people (Teipel et al., 2009). Annual income, occupation with the longest tenure in her/his life, and the occupation tenure were also included in the regression analysis because income and occupation were found to correlate significantly with MCI in a previous study (Kengsakul et al., 2015). The significance level was determined at *p* < 0.05. All statistical analyses were performed using IBM SPSS Statistics Version 20 (IBM Corp., Armonk, NY, United States). Data used for the analysis is provided in Supplementary Table S1.

## Results

As the population ages worldwide, the prevalence of cognitive disorders including MCI is increasing. To develop timely prevention and early treatment strategies by identifying biological factors, we investigated the relationship between dietary consumption of fish, brain structure, and MCI in cognitively normal subjects. The brain structure was assessed using neuroimaging-derived measures including GM-BHQ and FA-BHQ. Dietary consumption of fish was calculated using BDHQ, and MCI was assessed using the MPI of MCI Screen. MPI ranges from 0 to 100 and larger values indicates better performance. Descriptive statistics of subjects and correlation coefficients between scales are shown in Table 1. FA-BHQ scores correlated with Fish Intake (*r* = 0.304, *p* < 0.01) and MPI (*r* = 0.370, *p* < 0.01). In addition, GM-BHQ scores correlated with age (*r* = -0.411, *p* < 0.001), sex (*r* = 0.273, *p* < 0.05), BMI (*r* = -0.259, *p* < 0.05), and annual income (*r* = -0.340, *p* < 0.01), but not with Fish Intake (*r* = -0.014, *p* > 0.05) or MPI (*r* = 0.163, *p* > 0.05). Therefore, we decided to include the GM-BHQ score in the model as a control variable, but not as a main variable, in the following analyses.

**TABLE 1:** Descriptive statistics of subjects and correlations coefficients between scales.

Variable		Mean	SD	1	2	3	4	5	6	7	8	9	10	11	12	13	14	15	16	17	18
1	Age	47.026	7.129																		
2	Sex (male = 1, female = 2)	1.592	0.495	0.067																	
3	BMI	22.697	3.388	–0.100	-0.533***																
4	Length of education	14.763	1.688	−0.463***	−0.229*	0.205															
5	Annual income	6.355	3.595	0.130	−0.622***	0.313**	0.058														
6	Tenure	5.039	1.076	0.165	–0.170	0.051	–0.156	0.255*													
7	Management	0.053	0.225	0.099	−0.284*	0.341**	0.068	0.307**	0.102												
8	Special/Technical	0.224	0.419	0.047	–0.068	–0.038	–0.094	0.212	0.098	–0.127											
9	Office worker	0.355	0.482	0.180	0.225	−0.286*	–0.092	–0.143	0.127	–0.175	-0.398***										
10	Sales	0.132	0.340	–0.100	0.006	0.028	–0.015	0.059	–0.160	–0.092	–0.209	−0.289*									
11	Service	0.145	0.354	–0.107	0.113	–0.014	–0.031	–0.188	–0.085	–0.097	–0.221	−0.305**	–0.160								
12	Security	0.013	0.115	–0.017	–0.139	0.009	0.085	0.118	0.104	–0.027	–0.062	–0.086	–0.045	–0.048							
13	Production	0.026	0.161	–0.093	–0.031	0.129	0.121	–0.039	–0.160	–0.039	–0.088	–0.122	–0.064	–0.068	–0.019						
14	Transportation	0.013	0.115	–0.163	–0.139	0.046	0.223	0.021	–0.112	–0.027	–0.062	–0.086	–0.045	–0.048	–0.013	–0.019					
15	Unemployed	0.039	0.196	–0.106	–0.107	0.232*	0.150	−0.266*	–0.071	–0.048	–0.109	–0.150	–0.079	–0.083	–0.023	–0.033	–0.023				
16	GM-BHQ	99.792	5.578	−0.411***	0.273*	−0.259*	0.046	−0.340**	–0.109	–0.007	–0.093	0.061	–0.211	0.175	–0.094	0.126	0.063	0.022			
17	FA-BHQ	96.477	3.429	–0.114	0.149	–0.071	0.081	0.029	–0.066	0.068	0.045	–0.040	0.149	–0.144	0.056	0.055	–0.077	–0.108	0.153		
18	Fish Intake	41.921	26.027	0.025	–0.156	0.162	0.091	0.220	–0.062	–0.010	–0.021	0.022	0.125	–0.066	0.043	–0.012	–0.107	–0.047	–0.014	0.304**	
19	MPI	73.510	7.434	−0.546***	0.068	0.089	0.412***	–0.189	–0.078	0.032	0.145	–0.192	–0.003	0.063	0.013	–0.114	0.042	0.077	0.163	0.370**	0.125

n = 76; *p < 0.05; **p < 0.01; ***p < 0.001. BMI, body mass index; FA-BHQ, fractional-anisotropy brain healthcare quotient; GM-BHQ, gray-matter brain healthcare quotient; MPI, memory performance index.

Table 2 shows the results of regression analyses. In Step 1, none of the control variables significantly correlated with FA-BHQ scores. In addition, in the model with MPI as the dependent variable, age (*R* = 0.695, *b* = -0.483, *p* < 0.001), length of education (*R* = 0.695, *b* = 0.275, *p* = 0.018), and special/technical occupation (*R* = 0.695, *b* = 0.259, *p* = 0.021) significantly correlated with MPI, indicating that MPI is higher in those with younger ages, high educational backgrounds, and special/technical occupation than in those with older ages, low educational backgrounds, and other occupations. In Step 2, Fish Intake (*R* = 0.493, *b* = 0.309, *p* = 0.016) was significantly associated with higher FA-BHQ scores, and FA-BHQ scores (*R* = 0.760, *b* = 0.336, *p* < 0.001), Fish Intake (*R* = 0.720, *b* = 0.206, *p* = 0.042), and annual income (*R* = 0.760/720, b = -0.304/-0.299, *p* = 0.025/0.039) were significantly associated with higher MPI. In Step 3, FA-BHQ scores (*R* = 0.766, *b* = 0.301, *p* = 0.003) were significantly associated with MPI, but Fish Intake (*R* = 0.766, *b* = 0.113, *p* = 0.251) was not.

**TABLE 2:** Multiple regression analysis of FA-BHQ, Fish Intake, and MPI.

	FA-BHQ				MPI							
	Step 1		Step 2		Step 1		Step 2				Step 3	
	β^a,b^	*p*-value	β^a,b^	*p*-value	β^a,b^	*p*-value	β^a,b^	*p*-value	β^a,b^	*p*-value	β^a,b^	*p*-value
**Demographic variables**												
Age	–0.039	0.811	–0.096	0.542	–0.483	< 0.001***	–0.470	< 0.001***	–0.521	< 0.001***	–0.492	< 0.001***
Sex (male = 1, female = 2)	0.277	0.132	0.291	0.101	0.142	0.324	0.049	0.713	0.151	0.281	0.064	0.634
BMI	0.021	0.894	–0.050	0.748	0.098	0.433	0.091	0.425	0.051	0.681	0.066	0.569
Length of education	0.111	0.445	0.067	0.633	0.275	0.018*	0.238	0.026*	0.246	0.031*	0.226	0.035*
Annual income	0.161	0.380	0.087	0.627	–0.249	0.088	–0.304	0.025*	–0.299	0.039*	–0.325	0.017*
Tenure	–0.035	0.789	0.006	0.960	0.042	0.684	0.054	0.568	0.069	0.493	0.067	0.475
Management	0.105	0.460	0.171	0.222	0.168	0.137	0.133	0.199	0.212	0.060	0.160	0.131
Special/Technical	0.085	0.542	0.111	0.409	0.259	0.021*	0.230	0.024*	0.276	0.012*	0.243	0.018
Sales	0.176	0.215	0.150	0.271	0.012	0.914	–0.047	0.645	–0.005	0.961	–0.051	0.621
Service	–0.126	0.353	–0.098	0.453	0.038	0.720	0.081	0.411	0.057	0.585	0.086	0.378
Security	0.095	0.442	0.090	0.448	0.035	0.721	0.003	0.975	0.031	0.740	0.004	0.962
Production	0.033	0.795	0.067	0.591	–0.161	0.116	–0.172	0.066	–0.139	0.165	–0.159	0.091
Transportation	–0.079	0.534	–0.024	0.843	–0.048	0.628	–0.022	0.811	–0.012	0.903	–0.005	0.959
Unemployed	–0.049	0.733	–0.022	0.871	–0.049	0.660	–0.033	0.747	–0.032	0.772	–0.025	0.806
GM-BHQ	0.191	0.226	0.115	0.457	–0.094	0.450	–0.158	0.169	–0.145	0.241	–0.179	0.123
**Main variables**												
FA-BHQ							0.336	< 0.001***			0.301	0.003**
Fish Intake			0.309	0.016*					0.206	0.042*	0.113	0.251
*R*	0.404	0.693	0.493	0.308	0.695	< 0.001***	0.760	< 0.001***	0.720	< 0.001***	0.766	< 0.001***
*R*^2^	0.163		0.243		0.482		0.577		0.518		0.586	

n = 76; *p < 0.05; **p < 0.01; ***p < 0.001. ^*a*^Standardized regression coefficient. ^*b*^Independent variables were selected using the forced entry method. Step1 includes control variables only. Step 2 includes control variables and either FA-BHQ or Fish Intake. Step3 includes all the variables.

In summary, Fish Intake had a positive correlation with FA-BHQ scores after adjusting for demographic information. Fish Intake or FA-BHQ scores had a positive correlation with MPI after adjusting for demographic information. In addition, FA-BHQ scores had a higher positive correlation with MPI than Fish Intake did.

**FIGURE 1:**
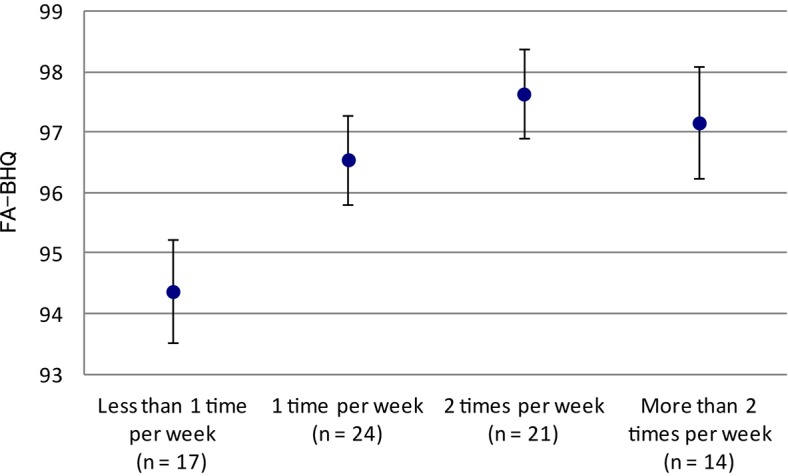
Fish intake frequency and FA-BHQ. The graph shows mean and standard error.

We next examined the appropriate frequency of fish intake in Tables 3, 4. Figure 1 is drawn using the information appeared in Table 3. Multiple comparison of the adjusted-mean FA-BHQ scores with analysis of covariance (ANCOVA) revealed significant differences between “less than 1 time per week” and “2 times per week” (*p* = 0.005) and between “less than 1 time per week” and “more than 2 times per week” (*p* = 0.034), consistent with the result of regression model Step 2 (with FA-BHQ as the dependent variable) in Table 2. In addition, multiple comparisons using adjusted-mean MPI scores with ANCOVA found significant differences between “less than 1 time per week” and “more than 2 times per week” (*p* = 0.037), consistent with the result of regression model Step 2 (with MPI as the dependent variable) in Table 2. However, no significant differences were found if FA-BHQ scores were used as control variables, consistent with the result of regression model Step 3 (with MPI as the dependent variable) in Table 2. Further, multiple comparison of the non-adjusted mean FA-BHQ scores using one-way analysis of variance (ANOVA) with the Scheffe’s *post hoc* test revealed significant differences between “less than 1 time per week” and “2 times per week” (*p* = 0.017), but not between “less than 1 time per week” and “more than 2 times per week” (*p* = 0.152). However, multiple comparisons of the non-adjusted mean scores of MPI using ANOVA with the Scheffe’s *post hoc* test showed no significant differences.

**TABLE 3:** Fish intake frequency and FA-BHQ.

	Fish intake frequency	Number of subjects	Non-adjusted mean^c^	Adjusted mean^d^	*p*-value of multiple comparisons
0	Less than 1 time per week	17	94.336 ± 3.591	94.369	
1	1 time per week	24	96.451 ± 3.010	96.552	0.070^0–1^
2	2 times per week	21	97.842 ± 3.251	97.641	0.005^0–2^**, 0.313^1–2^
3	More than 2 times per week	14	97.074 ± 3.208	97.163	0.034^0–3^*, 0.613^1–3^, 0.693^2–3^

n = 76; *p < 0.05; **p < 0.01; ***p < 0.001. ^*c*^ non-adjusted for any demographic variables ^*d*^ adjusted for age, sex, BMI, length of education, annual income, tenure, occupation, and GM-BHQ by ANCOVA method. ^0–1^ p-value derived from comparison between “less than 1 time” and “1 time” by ANCOVA method. ^0–2^ p-value derived from comparison between “less than 1 time” and “2 times” by ANCOVA method. ^0–3^ p-value derived from comparison between “less than 1 time” and “more than 2 times” by ANCOVA method. ^1–2^ p-value derived from comparison between “1 time” and “2 times” by ANCOVA method. ^1–3^ p-value derived from comparison between “1 time” and “more than 2 times” by ANCOVA method. ^2–3^ p-value derived from comparison between “2 times” and “more than 2 times” by ANCOVA method.

**TABLE 4:** Fish intake frequency and MPI.

	Fish intake frequency	Number of subjects	Non-adjusted mean^c^	Adjusted mean^d^	p-value of multiple comparisons
0	Less than 1 time per week	17	72.764 ± 5.883	70.875	
1	1 time per week	24	73.387 ± 7.238	74.722	0.068^0–1^
2	2 times per week	21	73.516 ± 8.845	72.810	0.329^0–2^, 0.312^1–2^
3	More than 2 times per week	14	74.617 ± 7.814	75.681	0.037^0–3^*, 0.650^1–3^, 0.179^2–3^

n = 76; *p < 0.05; **p < 0.01; ***p < 0.001. ^*c*^ non-adjusted for any demographic variables ^*d*^ adjusted for age, sex, BMI, length of education, annual income, tenure, occupation, and GM-BHQ by ANCOVA method. ^0–1^ p-value derived from comparison between “less than 1 time” and “1 time” by ANCOVA method. ^0–2^ p-value derived from comparison between “less than 1 time” and “2 times” by ANCOVA method. ^0–3^ p-value derived from comparison between “less than 1 time” and “more than 2 times” by ANCOVA method. ^1–2^ p-value derived from comparison between “1 time” and “2 times” by ANCOVA method. ^1–3^ p-value derived from comparison between “1 time” and “more than 2 times” by ANCOVA method. ^2–3^ p-value derived from comparison between “2 times” and “more than 2 times” by ANCOVA method. ANCOVA, analysis of covariance.

**FIGURE 2:**
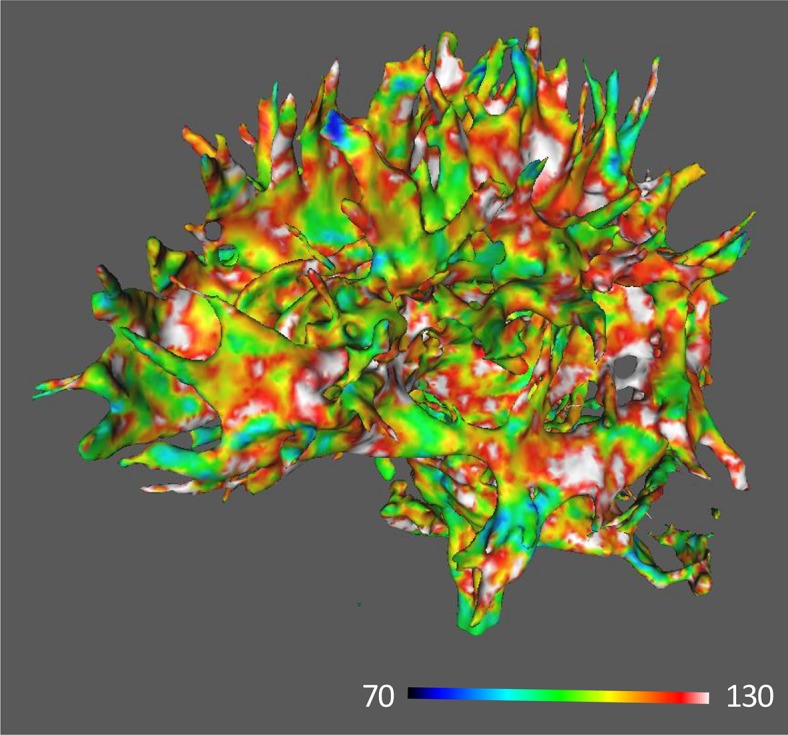
MRI image of the highest FA-BHQ score.

**FIGURE 3:**
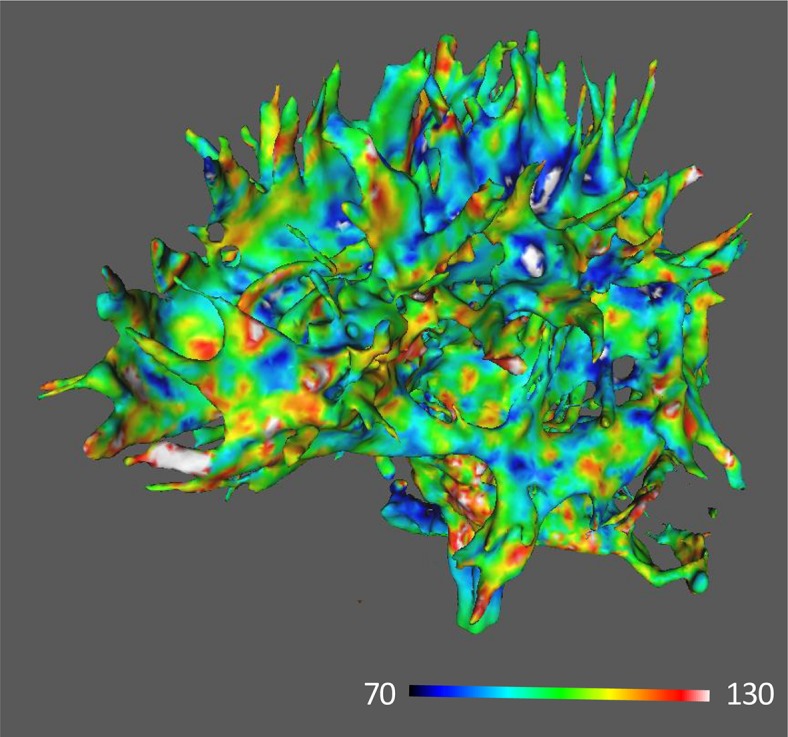
MRI image of the lowest FA-BHQ score.

Figures 2, 3 show images of MRI for subjects with the highest and the lowest scores of FA-BHQ, respectively.

## Discussion

As the population ages worldwide, the prevalence of cognitive disorders, including MCI, is growing (Alzheimer’s Disease International, 2015). MCI appears in 10–20% of adults aged 65 years and older (Langa and Levine, 2014) and is generally referred to as an intermediate stage between normal cognitive aging and dementia (Vega and Newhouse, 2014). To develop timely prevention and early treatment strategies, we investigated the relationship between dietary consumption of fish and the brain structure as well as between the brain structure and MCI in cognitively normal subjects. The brain structure was assessed using neuroimaging-derived measures including GM-BHQ and FA-BHQ, which are approved as the international standard (H.861.1) by ITU-T. Dietary consumption of fish was calculated using BDHQ (Sasaki et al., 2000), and MCI was assessed using the MPI of MCI Screen (the Medical Care Corporation). MPI ranges from 0 to 100 and larger values indicate better performance. The present study indicates that fish intake is positively associated with both FA-BHQ and MPI, and FA-BHQ scores are more strongly associated with MPI than fish intake.

One previous study found significant differences in the white matter integrity between MCI patients with and without cerebral amyloidopathy, indicating that alteration in white matter integrity can serve as a potential biomarker of MCI (Lee et al., 2017). In addition, several studies indicate that WML is associated with cognitive decline and incident dementia (Bokura et al., 2006; Buyck et al., 2009; Debette et al., 2010. Debette and Markus, 2010). Further, longitudinal studies reveals that WM DTI indices, including FA, can be used to predict cognitive decline and medial temporal lobe atrophy in subjective cognitive impairment (SCI) and MCI patients, indicating that DTI-derived information can be used as the predictor of conversion from MCI to AD (Selnes et al., 2013). Moreover, lower DHA levels in red blood cells are associated with greater WML volumes (Bowman et al., 2012; Tan et al., 2012), and daily fish oil supplementation has been found to decrease WMLs in cognitively normal people (Witte et al., 2013). In addition, it has been demonstrated that a member of the major facilitator superfamily, Mfsd2a (previously an orphan transporter), is the major transporter for DHA uptake into the brain (Nguyen et al., 2014).

The present results were derived from healthier and younger participants than those in most other studies. Our findings are in line with those in previous studies, but our study further found that the whole brain integrity measured by the FA-BHQ might be involved in the interaction between fish intake and MCI in healthy people. In other words, FA-BHQ may be used to identify people at high risk of MCI to provide the proper intervention. The findings in our study suggest that people with a good whole brain health condition, measured by FA-BHQ, tend to have lower risks of MCI. Although some previous studies found a direct effect of fish intake on prevention of MCI, the findings were limited and inconsistent (Zeng et al., 2017). Notably, the present study indicates that broader approaches can be used to prevent MCI by focusing on not only dietary factors but also FA-BHQ scores.

Additionally, multiple comparisons showed that FA-BHQ scores were significantly different between “less than 1 time per week” and “2 times per week” as well as between “less than 1 time per week” and “more than 2 times per week” fish intakes. These results indicate that fish diet should be taken at least 2 times a week to maintain high FA-BHQ scores. As shown in Figure 1, FA-BHQ scores of “more than 2 times per week” were marginally lower than those of “2 times per week” because intake of fish dishes is more likely to be accompanied with salad oil consumption, thus negatively affecting appropriate balance between omega-6 and omega-3 fatty acids and increasing the risk for overweight/obesity and coronary heart disease (Simopoulos, 2008).

Our analysis showed no association between fish intake and GM-BHQ. The result contradicts with the finding in the study by Raji et al. (2014), which indicates that consuming fish dishes at least weekly is related to larger GM volumes in the brain areas responsible for memory and cognition in cognitively normal elderly individuals. The discrepancy may result from the fact that many factors, including not only biological but also sociological factors, affect GM-BHQ, as shown in our previous studies (Nemoto et al., 2017; Kokubun et al., 2018; Kokubun and Yamakawa, 2019). In this sense, our findings are in line with another finding that although the brain volume was significantly related to fish consumption, it was not significantly associated with intake of plasma omega-3 fatty acids, suggesting that lifestyle factors accompanied by dietary intake of fish can also affect GM-BHQ (Raji et al., 2014).

Our study showed no association between GM-BHQ and MPI. This result is in line with that in a previous study, which indicates that WM tract degeneration is prominent in SCI and MCI patients and is at least in part independent of overlying gray matter atrophy (Selnes et al., 2012). In addition, Agosta et al. (2014) found that compared with healthy controls and cognitively unimpaired Parkinson’s disease patients, patients with Parkinson’s disease and MCI showed WM abnormalities in the anterior superior corona radiata, genu, and corpus callosum and the anterior inferior fronto-occipital, uncinate, and superior longitudinal fasciculi bilaterally, although no GM atrophy was found (Agosta et al., 2014).

This study has two limitations. First, the association of brain health with actual diseases was not examined in this study, and the illustration of the association of brain health with actual diseases may have improved the validity of the results. Second, the sample size was small in this study, and studies with a larger sample size may have increased the generalizability of the results. Therefore, future studies are needed to explore the relationship between FA-BHQ and actual diseases using larger sample sizes to discern the mechanisms.

## Data Availability Statement

All datasets generated for this study are included in the article/Supplementary Material.

## Ethics Statement

The studies involving human participants were reviewed and approved by the Ethics Committees of Kyoto University. The patients/participants provided their written informed consent to participate in this study.

## Author Contributions

KK wrote the main manuscript text and prepared the figures and tables. KN did MRI data analysis including GM- and FA-BHQ calculation. YY was responsible for the conceptualization, data curation, funding acquisition, and project administration. All authors reviewed and edited the manuscript.

## Conflict of Interest

YY was employed by NTT Data Institute of Management Consulting, Inc. The remaining authors declare that the research was conducted in the absence of any commercial or financial relationships that could be construed as a potential conflict of interest.

## Supplementary Material

TABLE S1 Data used for regression analyses.
